# Uterine cytokine profiles after low-molecular-weight heparin administration are associated with pregnancy outcomes of patients with repeated implantation failure

**DOI:** 10.3389/fendo.2022.1008923

**Published:** 2022-12-08

**Authors:** Zhihong Niu, Mingjuan Zhou, Lan Xia, Shen Zhao, Aijun Zhang

**Affiliations:** Department of Obstetrics and Gynecology, Ruijin Hospital, Shanghai Jiao Tong University School of Medicine, Shanghai, China

**Keywords:** cytokine, low-molecular-weight heparin, pregnancy, repeated implantation failure, embryo transfer

## Abstract

**Introduction:**

Low molecular-weight heparin (LMWH) plays a role in repeated implantation failure (RIF), but outcomes are controversial. LMWH can potentially modulate local immune responses associated with the establishment and maintenance of pregnancy. The study aimed to explore the effects of LWMH in uterine inflammatory cytokine profiles and pregnancy outcomes of patients with repeated implantation failure (RIF) but without thrombophilia.

**Methods:**

We compared clinical characteristics and reproductive outcomes among 326 patients with RIF, but not thrombophilia, undergoing frozen embryo transfer (FET) cycle with or without LMWH treatment. Endometrium secretions were aspirated from both groups after 3 days of progesterone administration before and after LMWH treatment. Cytokine mRNA expression was analyzed in primary endometrial cells in vitro.

**Results:**

The clinical and ongoing pregnancy rates did not significantly differ between the groups (31.5% vs. 24.4%, p = 0.15; 29.6% vs. 20.7%, p = 0.06). Concentrations of IL-6 and granulocyte-colony stimulating factor (G-CSF) in uterine secretions were significantly increased in the LWMH group, regardless of pregnancy outcomes (P < 0.05). And, in all patients treated with LWMH, those of secreted IL-6, IL-15 and G-CSF were significantly increased in pregnant group (P < 0.05). The expression of mRNA for G-CSF and IL-6 was significantly increased in human endometrial stromal cells in vitro (P < 0.05) after stimulation with LWMH (10 IU/mL).

**Conclusions:**

Uterine cytokine profiles after LMWH administration are associated with pregnancy outcomes and LMWH may be beneficial for patients with three implantation failures who do not have coagulation disorders.

## Introduction

Repeated implantation failure (RIF) is mainly defined as the inability to achieve a clinical pregnancy after three consecutive *in vitro* fertilization (IVF) attempts, involving the transfer of one or two high-quality embryos per cycle (1). Implantation failure has been associated with lifestyle habits (i.e. smoking and obesity), low quality of embryos, thrombophilia, uterine factors such as congenital uterine anomalies, endometrial polyps, and adnexal pathologies (i.e. hydrosalpinx). patients with RIF usually request further adjuvant therapies which generally can be grouped in four categories: uterine interventions (e.g. hysteroscopy, endometrial local injury); laboratory technologies and interventions(e.g. blastocyst culture, sequential embryo transfer, preimplantation genetic testing for aneuploidies); immunomodulatory or anticoagulant therapies (e.g. subcutaneous or intrauterine granulocyte colony stimulating factor administration, low-molecular-weight heparin,aspirin; prednisolone) and treatments enhancing endometrial receptivity(e.g. intramuscular growth hormone, endometrial receptivity array). Until now, the phenomenon of RIF presents a frustrating challenge for clinicians.

The administration of low-molecular-weight heparin (LMWH) is one of several methods that are currently being applied clinically to overcome implantation failure (2, 3). As an anticoagulant by facilitating the effects of antithrombin, LMWH might also modulate some of the mechanisms that underlie the successful implantation and penetration of developing embryos through their ability to interact with various adhesion molecules, growth factors, cytokines, and enzymes (4, 5). Heparin certainly improves pregnancy rates among women with repeated IVF failure and thrombophilia (6, 7). However, the improvement of pregnancy outcomes in patients without thrombophilia remains ambiguous. We previously assessed uterine cytokine profiles under different treatments or types of patients (8, 9). The present study aimed to define the effects of LMWH on uterine inflammatory cytokine profiles and its association with pregnancy outcomes of patients with RIF but without thrombophilia.

## Materials and methods

### Patients

We enrolled 393 women undergoing frozen embryo transfer at the Reproductive Medical Center of Ruijin Hospital affiliated to the Medical School, Shanghai Jiao Tong University. The inclusion criteria comprised: no ongoing pregnancy lasting beyond 10 weeks (dated from the day of ET) despite a cumulative total of at least six fresh or frozen embryos transferred on day 3 or day 5 of previous transfer cycles, age <38 years, body mass index (BMI) ≥ 19 and ≤ 25 kg/m^2^, and basal FSH ≤ 12 IU/L.

The exclusion criteria comprised a history of endocrine or metabolic disorders, ovarian cystectomy or oophorectomy, or pathology affecting the endometrial cavity and/or receptivity, and clinical and/or laboratory markers of hereditary or acquired thrombophilia and/or autoimmune disorders. The exclusion of thrombophilia and autoimmune disease was based on the two published meta-analysis (10, 11), including the laboratory test of protein C, protein S, antithrombin III activity, antiphospholipid antibodies, beta-2 glycoprotein I, Homocysteine, antinuclear antibodies and lupus antibody.

### Study design

**Figure 1:**
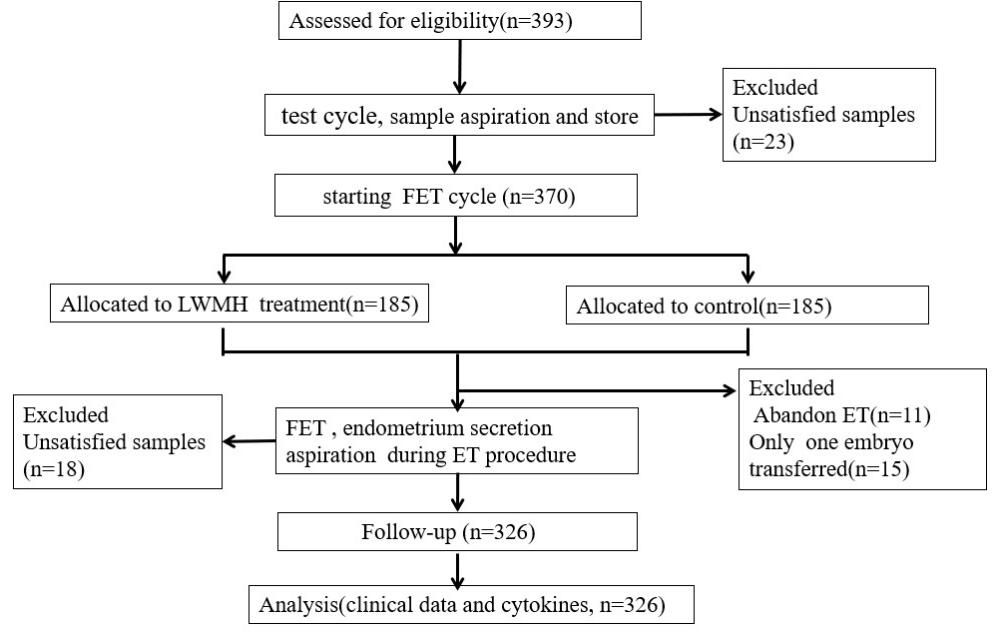
Flow chart of study . FET, frozen embryo transfer; LMWH, low molecular weight heparin.

All eligible participants underwent a test cycle with standard hormone replacement therapy (HRT) to prepare the endometrium before frozen embryo transfer (FET) cycles. Endometrial secretions aspirated on day 4 of progesterone administration were stored at -196°C. One or two months later, the patients were randomly assigned to receive either the standard HRT protocol with LMWH added (study group), or the standard HRT protocol alone (control group) from the initiation of the FET cycle. All patients provided written informed consent to participate after receiving a detailed explanation about the study and before the start of the treatment cycles. Endometrial secretions aspirated from all participants immediately before embryo transfer were stored at -196°C. **Figure 1** describes a flowchart of the study. Endometrial secretions were aspirated from 393 women with RIF during the test cycles and from 344 of them again during the FET cycles. We excluded 67 patients due to unsatisfactory samples (n = 41) and the absence of qualified embryos (n = 26).

### Endometrium preparation and FET

Briefly, incremental doses of oral estradiol (4–6 mg/day) started from days 2 or 3 of the menstrual cycle and continued for 12–20 days. Thereafter, the absence of folliculogenesis and an endometrial thickness of ≥8 mm was confirmed by transvaginal ultrasonography. Subsequently Vaginal progesterone (90 mg/day) was subsequently started, and FET proceeded on day 4 of progesterone administration.

The study group was administered with LMWH (enoxaparin sodium, Clexane, 4000 anti-Xa IU; Sanofi-Aventis, Paris, France) at a standard dose of 40 mg/0.4 mL/day starting on the day of progesterone administration. The patients subcutaneously self-administered the LMWH for 10 weeks thereafter if they tested positive for pregnancy, and discontinued if it was negative.

On day 4 of progesterone administration, morphologically selected embryos were thawed, then transferred *in utero.* The scores of embryos were evaluated according to our published standard (12) and only patients with 2 cleaved transferred embryos were included in the study. All patients received oral estradiol and vaginal progesterone supplement until 12 weeks of pregnant, or discontinued if test negative.

### Endometrial sampling and processing

Patients were placed in the lithotomy position, and then a speculum was inserted through the cervix, which was then cleansed. An embryo transfer catheter (CCD Laboratories, Paris, France) was transcervically introduced, and suction was gradually applied with a 2 ml syringe. The tips of the catheter were cut off and snap frozen in liquid nitrogen and stored at -80°C. Blood contamination with endometrial secretions can affect measurement of some cytokines; thus, samples that were moderately or severely contaminated were excluded from the analyses.

### Multiplex immunoassay

The samples of endometrial secretions were analyzed using multiplex immunoassays as we described (8). According to a published study (13), key soluble implantation regulators were identified as candidate mediators for inclusion in the assays. Mediators without appropriate antibodies or having cross interference were excluded. The final panel included interleukins (IL)-1b, IL-2, IL-4, IL-5, IL-6, IL-7, IL-8, IL-10, IL-12, IL-13, IL-15, IL-17, IL-18, leukemia inhibitory factor (LIF), tumor necrosis factor-α (TNF-α), interferon-γ (IFN-γ), granulocyte macrophage-colony stimulating factor (GM-CSF), granulocyte-colony stimulating factor (G-CSF), vascular endothelial growth factor (VEGF), macrophage inhibitory protein-1β (MIP-1b), and monocyte chemotactic protein-1 (MCP-1).

### Primary endometrium cell isolation and culture

We used primary human endometrial epithelial (HEEC) and stromal (HESC) cells as study models *in vitro*. The isolation procedure was based on a previous protocol (14) with slight modifications. Briefly, both primary cell types were isolated from fresh endometrial biopsies obtained from six women just following endometrial secretions aspiration in test cycles. The tissues were minced and digested, then primary stromal and epithelial cells were isolated, seeded on dishes in Dulbecco’s modified Eagle’s medium (DMEM) Complete Medium/F12 containing 1% (v/v) penicillin/streptomycin (Thermo Fisher Scientific Inc., Waltham, MA, USA) and 10% (v/v) fetal bovine serum (Thermo Fisher), and incubated at 37°C under a 5% CO_2_ atmosphere for 48 h for RNA isolation.

### RNA isolation and RT-qPCR

After immunocytochemically verifying the cells, human endometrial epithelial cells (HEEC) and stromal cells (HESC) were incubated with low (1 IU/mL) and high (10 IU/mL) concentrations of LMWH (Invitrogen, Carlsbad, CA, USA) for 48 h, and then extracted RNA using Trizol. The RNA was reverse transcribed using a cDNA Reverse Transcription Kit (Toyobo, Japan) and cDNA was synthesized using PrimeScript RT Master Mix (RR036A Takara Bio Inc., Kusatsu, Japan). The cDNA was stored at −20°C. The cDNA was reverse-transcribed using RT-qPCR with SYBR Premix Ex Taq kit (RR420A; Takara). Each reaction included an initial denaturation at 95°C for 30 min, 40 amplification cycles at 95°C for 5 s and annealing at 60°C for 34 s. Gene expression was normalized to that of glyceraldehyde-3-phosphate dehydrogenase (GAPDH). Fold change (FC) was calculated according to the 2^–ΔΔCt^ method and values with P < 0.05 were considered statistically significant. All cell samples were analyzed in triplicate.

### Pregnancy outcome measures

The primary outcome measure was ongoing pregnancy rates. Secondary outcome measures comprised biochemical (positive β-hCG), and clinical pregnancy rates. Ongoing pregnancy rates were defined as the presence of at least one fetal heart pulse on ultrasound beyond 20 weeks and clinical pregnancy was defined as ultrasound confirmation of a gestational sac at 4 weeks after embryo transfer. Outcome measures were not changed after the trial commenced.

### Sample size calculation

The sample size was calculated using Stat version 13 (StataCorp, College Station, TX). The pregnancy rate of patients with RIF in our center has been rather low (<20%). Considering the scenario where intervention increases clinical pregnancy rates from 20% to 40%, then 100 participants would be required in each arm to provide a significance level of 0.05 and a power of 0.8 in the analyses of outcomes.

### Statistical analysis

Non-normally distributed data regarding uterine cytokine profiles were log-transformed before analysis. Continuous variables are expressed as medians with ranges and were compared using nonparametric Mann–Whitney U-tests. Proportions of categorical variables were compared using Chi-square and Fisher exact tests. All tests were two-sided and values were considered statistically significant at p < 0.05. All data were statistically analyzed using SPSS 26.0 (IBM Corp., Armonk, NY, USA).

## Results

### Baseline and cycle characteristics

The final analysis included 326 patients. Age, BMI, ovarian reservation, and duration of infertility did not significantly differ between patients treated with or without LMWH. The median numbers of previous implantation failures were 4 (3–6) in both groups and none of the patient smoked cigarettes. **Table 1** shows the clinical characteristics of the groups.

**Table 1:** Baseline and demographic characteristics of the participants.

	LMWH (N = 162)	Control (N = 164)
Age (y)	33 (27–38)	32 (26–38)
BMI (kg/m^2^)	22.4 (20.6–25.4)	21.9 (19.1–25.7)
Basal FSH (IU/L)	8.2 (6.5–11.2)	8.7 (6.3–11.7)
AFC	8 (6–15)	9 (5–15)
Duration of infertility (y)	5 (1–8)	4 (1–9)
Smoking (n, %)	0	0
No of previous implantation Failures	4 (3–6)	4 (3–6)
Primary subfertility (n, %)	122,75.3%	129,78.7%
The history of spontaneous abortion (n, %)	10,6.2%	12,7.3%

All values are P > 0.05. AFC, antral follicle count; BMI, body mass index; FSH, follicle stimulating hormone.

### Treatment outcomes

**Table 2** summarizes the pregnancy outcomes.

**Table 2:** Summary of treatment and pregnancy outcomes of LMWH and control groups.

	LMWH (N = 162)	Control (N = 164)	p
Endometrium thickness (mm)	9.2 ± 1.3	9.4 ± 1.1	0.32
Hormone level on ET day			
E2 (pg/ml)	122.6 ± 33.7	133.9 ± 31.2	0.19
P (ng/ml)	7.2 ± 1.5	8.1 ± 1.3	0.11
Embryos transferred (n)	2.0	2.0	> 0.99
Average scores of transferred embryos	7.1 ± 1.2	7.3 ± 1.5	0.14
Positive pregnancy test (n,%)	55 (34.0%)	45 (27.4%)	0.20
Clinical pregnancy (n,%)	51 (31.5%)	40 (24.4%)	0.15
Ongoing pregnancy (n,%)	48 (29.6%)	34 (20.7%)	0.06
Implantation (n,%)	55 (17.0%)	42 (12.8%)	0.14
Miscarriage (n,%)	3 (5.9%)	6 (15.0%)	0.21
LMWH side effects	0	0	> 0.99
LMWH infection	0	0	> 0.99

All patients were transferred with two cleaved embryos on day3 of the FET cycle. Cycle characteristics, including endometrium thickness, hormone level, and average scores of transferred embryos did not significantly differ between the groups. Rates of positive results in pregnancy tests (biochemical pregnancy) and clinical pregnancies were similar in both groups (34.0% vs. 27.7%, p = 0.2, and 31.5% vs. 24.4%, p = 0.15).

Miscarriage and ongoing pregnancy rates were also similar between the control and LWMH groups (5.9% vs. 15.0%, p = 0.21 and 29.6% vs. 20.7%, p = 0.06, respectively) although the ongoing pregnancy rate tended to increase in the LWMH group. All miscarriages occurred between 7 and 10 weeks of gestation and among the 9 patients of miscarriage, two and four patients in the control and treatment groups, respectively, had spontaneous abortion at least once. None of the patients reported discomfort or side effects.

### Endometrial cytokine profiles

**Table 3** shows a comparison of the medians and 25^th^ and 75^th^ percentiles of concentrations of each mediator in the endometrium at the test and FET cycles between the two groups. Endometrium cytokine concentrations did not significantly differ between the test and FET cycles in the control group. Cytokines that significantly differed between the two cycles in the LWMH group comprised IL-6 (P < 0.05) and G-CSF (P < 0.05). Concentrations of IL-8, IL-10, IL-15, TNF-α, GM-CSF, VEGF, MIP-1b and MCP-1, differed in the LWMH group with P value >0.05 but < 0.1

**Table 3:** Table III. Comparison of uterine cytokine concentrations in endometrial secretions between LMWH and control groups.

Cytokine concentrations (pg/mg total protein)				
	LMWH (N = 162)		Control (N=164)	
	Test cycle	FET cycle	Test cycle	FET cycle
IL-1b	19.5 (13.6;30.9)	17.2 (11.5;29.6)	17.6 (11.2;31.9)	19.3 (10.2;31.5)
IL-2	16.3 (10.8; 26.1)	15.6 (11.2;28.9)	18.9 (9.2;22.3)	17.1 (10.2;25.6)
IL-4	11.1 (7.8;16.8)	12.1 (10.2;20.5)	10.2 (8.1;17.2)	11.9 (9.1;18.2)
IL-5	12.3 (8.8;19.6)	14.2 (7.2;22.8)	10.4 (8.2;18.5)	12.1 (7.2;23.5)
IL-6^a^	18.1 (12.3;31.7)	28.5 (19.8;45.3)	16.2 (11.9;32.6)	18.3 (10.7;33.8)
IL-7	13.3 (8.7;19.9)	11.2 (9.6;20.3)	12.4 (8.5;19.9)	12.9 (8.1;20.3)
IL-8^b^	396.6 (197.1;892.2)	498.2 (244.6; 887.1)	371.2 (200.6;906.3)	369.3 (191.3;813.4)
IL-10	27.3 (19.4;44.7)	25.3 (20.8;45.6)	24.2 (18.5;42.9)	26.9 (17.6;50.3)
IL-12	13.8 (10.2;22.4)	12.1 (9.8;22.4)	15.8 (11.3;20.9)	16.1 (10.9;23.2)
IL-13	9.9 (7.0;15.3)	11.2 (6.8;16.2)	11.2 (7.8;16.8)	12.8 (7.9;18.4)
IL-15^b^	10.3 (6.4;18.2)	13.4 (7.9;21.2)	11.3 (5.8;19.4)	10.8 (6.3;18.9)
IL-17	17.3 (11.6;28.3)	15.2 (10.9;27.2)	18.1 (10.2;29.9)	16.2 (9.2;26.9)
LIF	79.1 (59.2;115.6)	88.9 (62.3;130.8)	69.2 (55.4;120.9)	78.7 (60.9;129.6)
IL-18	9.5 (7.0;15.2)	11.4 (8.0;18.7)	10.1 (7.2;14.1)	12.0 (7.5;19.6)
MCP-1^b^	419.0 (150.1;866.7)	302.3 (198.3;812.4)	388.2 (191.3;992.3)	416.8 (167.2;813.3)
MIP-1b^b^	125.8 (76.1;227.0)	100.3 (77.2;250.1)	119.3 (56.2;238.9)	131.4 (62.9;277.1)
TNF-a^b^	17.6 (13.3;30.2)	13.4 (10.2;25.3)	16.2 (12.9;33.2)	18.1 (11.8;34.1)
VEGF^b^	13.2 (8.4;22.7)	16.2 (8.9;23.8)	14.9 (9.2;25.4)	15.3 (9.1;27.2)
G-CSF^a^	90.5 (53.6;117.4)	133.6 (62.2;160.3)	100.3 (50.2;128.4)	112.6 (59.9;138.7)
GM-CSF^b^	18.2 (13.4;31.9)	14.1 (11.2;32.9)	17.1 (11.8;32.4)	16.2 (9.8;33.7)
IFN-g^b^	18.1 (12.3;28.9)	22.1 (11.2;29.6)	19.4 (14.2;30.7)	17.9 (12.8;31.5)

Values are shown as medians (25^th^; 75^th^ percentiles). Two-tailed paired t-tests on log transformed data. ^a^p < 0.05, ^b^p < 0.1.

### Intra-patient comparison of endometrial cytokines between two FET cycles

**Figure 2:**
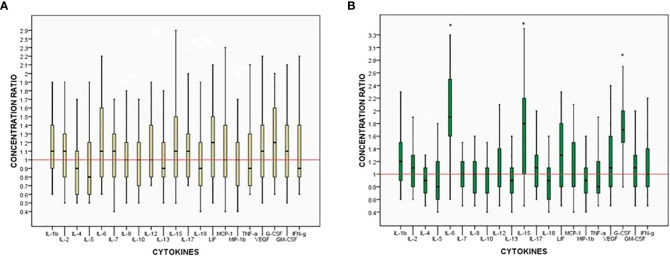
Ratios of intra-patient endometrium cytokine concentrations between two cycles. Box: median, 25%–75% interquartile range (IQR). Whiskers 10%–90% IQR. **(A)** Non-pregnant women. **(B)** Pregnant women. *p<0.05.

Following the changes in the cytokine profiles induced LWMH treatment, we compared the cytokine concentrations between test and FET cycles in each patient of the LWMH group to determine the relationship between cytokine changes and pregnancy outcomes. **Figure 2** depicts the endometrial cytokine concentration ratios between the two cycles in individual patients based on whether they became clinically pregnant. The expression of all tested soluble mediators did not differ significantly between pregnant and non-pregnant women (**Figure 2A**), whereas the concentrations of IL-6, IL-15, and G-CSF were all significantly higher in pregnant patients treated with LWMH (P < 0.05; **Figure 2B**)

### Changes in cytokine expression induced by LWMH in primary endometrial cells

**Figure 3:**
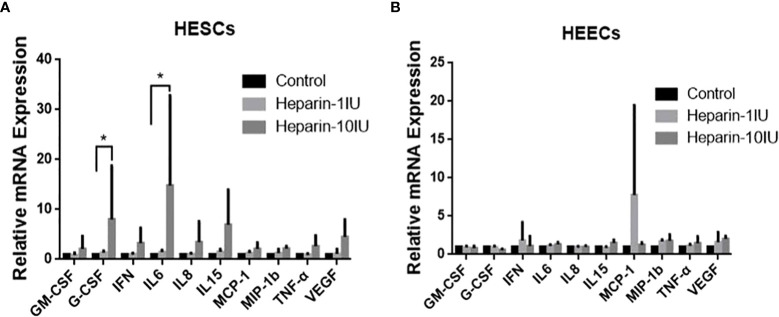
Cytokine mRNA levels are altered in HESC and HEEC incubated with LWMH for 48h. Data are presented as means ±SEM and *P<0.05. **(A)** Human endometrial stromal cells. **(B)** Human endometiral epithelial cells.

We investigated the influence of LWMH on cytokine expression in primary endometrium cells *in vitro*. Because the results *in vivo* indicated that LWMH altered the concentrations of IL-6, IL-8, IL-10, IL-15, TNF-α, GM-CSF, G-CSF, VEGF, MIP-1b and MCP-1, we measured their levels of mRNA expression of *in vitro*. **Figure 3A** shows that stimulation with LWMH (10 IU/ml) significantly increased the mRNA expression of G-CSF and IL-6 in HESC (P < 0.05), whereas the expression of all other cytokines in HEEC did not obviously differ (P > 0.05; **Figure 3B**).

## Discussion

Although RIF has not been unanimously defined, it is characterized by failure to achieve pregnancy after multiple embryo transfers. The incomplete understanding of RIF and its diverse etiologies pose therapeutic challenges. When a good-quality embryo fails to result in pregnancy, the ability of the endometrium to allow effective embryo apposition and adhesion is frequently suspected. However, non-organic uterine aberrations are difficult to detect in the clinical setting. The active, and especially the innate immune systems, are important components of this process, and uterine immune disequilibrium could have a negative impact on embryo implantation. Several immunological abnormalities have been linked to RIF and recurrent pregnancy loss (RPL) (15–17).

LWMH can prevent thrombin-induced pregnancy loss in women with antiphospholipid antibodies (APA) (6, 7). Interaction studies between LWMH and implantation-related uterine factors have revealed potential benefits of LWMH during very early pregnancy (18, 19). However, whether LWMH can confer benefits on patients with RIF but no thrombophilia has remained unclear.

The clinical parameters in FET cycles revealed that FET with or without LMWH treatment did not result in statistically significant differences in clinical pregnancy, ongoing pregnancy, and miscarriage rates among 326 patients with three unsuccessful IVF/ICSI cycles enrolled in the study. Several recent trials have found a trend toward a positive effect of LWMH in women with RIF but no APS, but the evidence is still inconclusive (20, 21). According to a meta-analysis by Akhtar et al. (20) that included three RCT (386 women), analyses of fixed, but not random effects have linked LWMH to higher rates of live birth and pregnancy. However, only one of the three RCT studies has investigated women who had at least two failed IVF cycles. Drakakis et al. (22) conducted a multicenter cohort study included 230 women who had at least two failed fresh IVF/ICSI cycles. They found no statistically significant differences in clinical pregnancy and miscarriage rates between those who received LWMH and those who did not. The current and previously published findings indicate that the evidence supporting the routine administration of LWMH to RIF patients remains weak. Furthermore, it is still not recommended for general patients undergoing IVF.

For embryo attachment and invasion, a local immune biological reaction should be orchestrated and balanced. A pro-inflammatory environment is critical during the pre-implantation period, and uterine cytokines play a role as intercellular messengers in this immune remodeling. To the best of our knowledge, this was the first study to look at the effects of LWMH treatment on uterine secretion cytokines *in vivo*. Here, LWMH administration was found to be associated with increased uterine concentrations of IL-6 and G-CSF in RIF women. Furthermore, LWMH administration increased IL-8, IL-15, VEGF, IFN- and decreased MCP-1, MIP-1b, and TNF-a levels in uterine secretions. The most intriguing finding of the study was that LWMH administration significantly increased IL-6, IL-15, and G-CSF concentrations in pregnant patients but not in non-pregnant patients. We hypothesized that the variable role of LMWH in women with RIF is dependent on the individual uterine immune microenvironment and its response to LWMH stimulation, based on the variety of LWMH effects and their association with successful embryo implantation.

In immunology effects, IL-6 and IL-15 were generally acknowledged as pro-inflammatory cytokines. The mid-secretory phase of the cycle is when the concentration of IL-6 produced by glandular and luminal epithelial cells in human endometrium peaks, indicating that IL-6 plays a role in embryo implantation (23, 24). It was shown that IL-6 induces the production of metalloproteinases (MMP)-14, MMP-11, and leptin secretion to alter endometrial tissues to permit trophoblast invasion (25). Additionally, through controlling trophoblast cell proliferation, differentiation, and hCG synthesis, it is a crucial regulator of early placental development (26, 27). Insufficient local IL-6 may contribute to fetal loss, since IL-6 expression is reduced during the mid-secretory phase of endometrial of women prone to recurrent spontaneous abortion (RSA) (28). Consistent with the role of IL-6 in key reproductive events, IL-6 null mutant mice exhibit elevated fetal resorption and delayed parturition, while exogenous IL-6 can correct the fetal loss (29).

However, it should be noted that excessive IL-6 expression may negatively impact early pregnancy in a number of cell signaling pathways. In a recent study, we demonstrated that uterine IL-6 concentrations are elevated in adenomyosis patients and that this is associated with decreased endometrial receptivity (8). Women with endometriosis and unexplained infertility have been observed by other investigators to have higher endometrial IL-6 concentrations (30). As a result, the individualized uterine environmental setting of the woman should determine the beneficial effect of LMWH on embryo implantation and growth.

Interleukin-15, a different cytokine associated with LMWH therapy, is expressed in both epithelial and stromal cells, and its expression rises in the latter following decidualization (31). Studies conducted *in vitro* have demonstrated that IL-15 has a role in the endometrial recruitment of peripheral blood NK cells and subsequent differentiation into uterine NK cells (uNK cells) (32). Additionally, IL-15 is a potent stimulator of uNK cell activation and proliferation (33). Conclusions regarding stromal expression of IL-15 in RIF-affected women were contentious (34, 35). Actually, uNK cells have dual functions. While proper uNK cell activation is essential for optimal embryo implantation, in a Th1-dominant environment, uNK cells transform into cytotoxic killer cells that recognize and reject trophoblastic cells as non-self. A study by Lédée et al. (36) identified low IL-15/fibroblast growth factor-inducible molecule 14 (Fn-14) mRNA as a biomarker of insufficient uNK cell activation/maturation in RIF patients. This ratio was raised with higher pregnancy rates in the subsequent embryo transfer cycle following individualized care, such as endometrial local injury. The current study discovered that successful pregnancies in RIF women were associated with increased IL-15 secretion caused by LWMH medication, suggesting that LWMH may act as a possible immunological regulator in some RIF women.

Unlike IL-6 or IL-15, G-CSF usually plays role in the initiation and maintenance of pregnancy by temporarily suppressing immune response through its effects on lymphocytes, macrophages, and Th-2 type cells. Clinically, G-CSF was found to be more abundant in the placental villous tissues of normal pregnancy women than in those with RSA (37).. Gleicher et al. first described a potential benefit of G-CSF for women with thin endometrium who undergo frozen embryo transfer in 2014 (38). According to Kamath (39), G-CSF was associated with a significantly higher clinical pregnancy rate with a pooled risk ratio of 2.51 (95% CI, 1.36-4.63) in a RIF population compared to patients who received no intervention. However, a recent randomized controlled trial found that intrauterine G-CSF administration had no effect on pregnancy outcomes in RIF patients with normal endometrium (40). The discovery that LWMH stimulated uterine G-CSF secretion provides a new explanation and strategy for improving endometrial receptivity in RIF patients regardless of endometrial thickness.

*In vitro*, we investigated the effects of LWMH on cytokine production in HEEC and HESC. None of the candidate cytokines were affected by LWMH exposure at either 1 or 10 IU/mL in HEEC cells. G-CSF and IL-6 mRNA expression was significantly increased in HESC cells treated with 10 IU/mL LWMH for 48 hours. Although LWMH therapy increased uterine IL-15 secretion in pregnant patients, we were unable to replicate this effect in cultured cells *in vitro*.

The function of LWMH on cultured HEEC and HESC *in vitro* is subject to debate. LWMH is commonly described as an anti-inflammatory and immunoregulatory agent that inhibits NK cell cytotoxicity (41) or as a leukocyte recruitment and vascular adhesion inhibitor (42). For example, it has been reported that LMWH can inhibit TNF-induced IL-6 expression by interacting with the transcription factor nuclear factor of transcription (NF) B in human ESCs (43). In contrast to previous research, we discovered that LWMH increased the expression of IL-6, a pro-inflammatory cytokine, but had no effect on TNF-expression in HESC. Variations in the content of various LWMH, which is a mixture of polysulfated glycosaminoglycans, could explain this phenomenon. Spratte et al. found that the ability of LMWH to regulate cytokines in HESC depends on its molecular size (19).

Quao et al. (44) reported the upregulation of G-CSF induced by LWMH stimulation in basal cultures of human HEEC, which we confirmed in both vivo and *in vitro* experiments. Given the effects of G-CSF on trophoblast invasion, its increased expression may play a role in RIF patients’ embryo implantation. However, the elevated IL-15 levels in pregnant patients given LWMH were not confirmed *in vitro*, implying that the molecular mechanisms underlying LWMH’s influence on uterine cytokine signaling pathways are complicated.

The current study discovered unique uterine cytokine profiles in the presence of LMWH. We discovered that increased endometrial IL-6, IL-15, and G-CSF secretion caused by LMWH is linked to better pregnancy outcomes in RIF patients. Although clinical outcomes did not differ significantly between the LWMH and control groups, our findings suggested that LMWH may be beneficial for patients with three implantation failures who do not have coagulation disorders. The findings of this study suggest that large randomized placebo-controlled trials are needed to assess the role(s) of LMWH in the regulation of uterine local immunity *in vivo*. Furthermore, because the molecular weight, half-life, and activities of LMWH prepared using different methods vary, comparative studies are required to evaluate their ability to improve embryo implantation in women with RIF.

## Data availability statement

The original contributions presented in the study are included in the article/supplementary material. Further inquiries can be directed to the corresponding authors.

## Ethics statement

The studies involving human participants were reviewed and approved by The ethics committee of Ruijin Hospital. The patients/participants provided their written informed consent to participate in this study.

## Author contributions

ZN and AZ conceived and designed the experiments, and composed the paper. MZ performed the cell experiments and interpreted the results. LX and SZ participated in study execution. All authors participated in the revision and final approval of the manuscript.
